# Plasticity and evolved changes in mitochondrial physiology across skeletal muscles of high-altitude deer mice (*Peromyscus maniculatus*)

**DOI:** 10.1242/jeb.252523

**Published:** 2026-08-24

**Authors:** Ranim Saleem, Samuel G. Robichaud, Simon G. Lamarre, Jay F. Storz, Graham R. Scott

**Affiliations:** ^1^ Department of Biology, McMaster University, 1280 Main Street West, Hamilton, ON, Canada, L8S 4K1; ^2^ Département de Biologie, Université de Moncton, 18 avenue Antonine-Maillet, Moncton, NB, Canada, E1A 3E9; ^3^ School of Biological Sciences, University of Nebraska, Lincoln, NE 68588, USA

**Keywords:** High-altitude adaptation, Hypoxia, Acclimation, Mitochondrial respiratory capacity, Evolutionary physiology

## Abstract

Small mammals at high altitude face the challenge of maintaining high rates of thermogenesis to cope with cold temperatures despite low O_2_ availability to fuel aerobic metabolism. We examined how adjustments in mitochondrial physiology across skeletal muscles might help overcome this challenge in deer mice (*Peromyscus maniculatus*) native to high altitude. Mice from high- and low-altitude populations were born and raised in captivity, and adults were acclimated to warm normoxia or cold (5°C) hypoxia (∼12 kPa O_2_ for 6–8 weeks) in a full-factorial design. Mitochondrial respiration and reactive oxygen species (ROS) emission were measured in the diaphragm, vastus medialis, vastus lateralis and gluteus maximus, complemented by measurements of mitochondrial abundance by transmission electron microscopy. In general, acclimation to cold hypoxia increased mitochondrial volume density (in some cases due to a preferential enrichment of subsarcolemmal mitochondria), mitochondrial respiration and/or complex IV activity, and also reduced ROS emission (measured both *ex vivo* and *in vivo*) in multiple muscles. Overlaid upon these effects of acclimation, high-altitude mice exhibited greater mitochondrial respiratory capacity than low-altitude mice in the diaphragm, and greater respiratory capacity of complex IV in all muscles except the gluteus maximus. High-altitude mice also exhibited lower ROS emission or more pronounced reductions in ROS emission in some conditions. These findings suggest that plastic and evolved changes in mitochondrial physiology can help improve energy supply and mitigate oxidative stress in small mammals at high altitude.

## Teaser

**Highlighted Article:** Plastic and evolved changes in mitochondrial abundance, respiratory capacity, and ROS emission help improve cellular energy supply and mitigate oxidative stress across skeletal muscles in deer mice at high altitude.

## INTRODUCTION

Mitochondria are key determinants of cellular energy production and redox balance, and thus underlie the capacity of organisms for sustained aerobic activity and various other performance traits (Koch et al., 2021). Mitochondrial respiratory function can exhibit plastic responses to changing metabolic demands, as exemplified by the increases in mitochondrial content and oxidative phosphorylation capacity that occur in muscle in response to aerobic exercise training (Farhat et al., 2015; Porter et al., 2015; Zoladz et al., 2016; Granata et al., 2018; Meinild Lundby et al., 2018; Flensted-Jensen et al., 2021; Batterson et al., 2023). Mitochondrial abundance also varies across species, as illustrated by interspecific comparisons that have revealed strong positive correlations between whole-body mitochondrial content and aerobic capacity (*V̇*_O_2_,max_) (Hoppeler, 1990; Weibel et al., 2004; Jacobs and Lundby, 2013). Evolved variation in mitochondrial phenotypes among populations and species may be shaped by natural selection during the process of environmental adaptation (O'Brien and Mueller, 2010; Chung et al., 2017; Heine and Hood, 2020; Koch et al., 2021). However, it remains unclear in many contexts how plastic and evolved changes in mitochondrial physiology combine to shape aerobic performance and help organisms cope with environmental challenges.

Endotherms native to high altitude provide an opportune system for understanding the mitochondrial mechanisms for overcoming environmental stress. High-altitude environments are both hypoxic and cold, challenging the ability to maintain sufficient rates of mitochondrial ATP production to meet the high energetic demands of thermogenesis, locomotion, reproduction and various other fitness-relevant traits. Human studies have demonstrated plasticity in mitochondrial abundance and respiratory function in skeletal muscle at high altitude; however, the magnitude and direction of the responses have varied depending on the severity and duration of exposure and whether subjects maintained neutral energy balance (Kayser et al., 1991; Jacobs et al., 2012, 2013, 2016; Levett et al., 2012). In non-human mammals and birds, in which cold likely poses greater challenges to thermoregulation (especially in smaller species), high-altitude natives have often shown increased mitochondrial density and enhanced oxidative capacity in skeletal muscles (Mathieu-Costello et al., 1998; Dawson et al., 2016). Plasticity likely contributes to this variation, based on studies showing that cold acclimation can increase mitochondrial volume and oxidative capacity to support muscle shivering (Zaninovich et al., 2003; Mollica et al., 2005; Bal et al., 2017a,b; Mahalingam et al., 2020). However, comparisons of high- versus low-altitude taxa raised in common lab environments have revealed evolved increases in muscle mitochondrial volume and/or respiratory capacity (Scott et al., 2009; Mahalingam et al., 2017). Few previous studies have examined how the uniquely evolved (genetically based) features of high-altitude taxa combine with plasticity in response to cold hypoxia to shape mitochondrial respiratory function. Fewer studies have examined mitochondrial metabolism of reactive oxygen species (ROS) at high altitude, even though mitochondria are a major source of ROS within cells (Murphy, 2009) and high-altitude exposure can augment ROS generation and lead to oxidative stress (Dosek et al., 2007).

We sought to examine the evolution and plasticity of mitochondrial physiology in a range of skeletal muscles in the North American deer mouse (*Peromyscus maniculatus*). This species occupies a wide elevational range from near sea level to over 4300 m in the Rocky Mountains (Hock, 1964; Snyder et al., 1982; Natarajan et al., 2015). High-altitude populations of deer mice must sustain high metabolic rates to support thermogenesis in the wild (Hayes, 1989) and there is evidence for strong directional selection for increased thermogenic capacity (Hayes and O'Connor, 1999). High-altitude deer mice have responded to this strong selection pressure with evolved increases in thermogenic capacity under hypoxia (Cheviron et al., 2013; Tate et al., 2020). The underlying mechanism of this evolved difference appears to include a change in the oxidative phenotype of skeletal muscle. In studies of the gastrocnemius, a large hindlimb muscle involved in shivering and locomotion, high-altitude mice were observed to have greater capillarity and respiratory capacity in the muscle compared with low-altitude mice, which was driven by increased relative abundance of oxidative fibre types and increased volume density, respiratory capacity and O_2_ affinity of subsarcolemmal mitochondria (Lui et al., 2015; Mahalingam et al., 2017, 2020; Dawson and Scott, 2022). Hypoxia acclimation also reduced mitochondrial ROS emission, and in some cases the reduction was greater in high-altitude mice than in low-altitude mice (Mahalingam et al., 2017; Dawson and Scott, 2022). The evolution and plasticity of these mitochondrial phenotypes across other skeletal muscles remain largely unresolved (Garrett et al., 2024), but such information is needed to provide a broad appreciation of mitochondrial adaptation to high altitude. Here, we examined mitochondrial respiratory capacity, abundance and ROS emission in four skeletal muscles that exhibit a range of oxidative capacities, using a combination of high-resolution respirometry and fluorometry along with transmission electron microscopy (TEM). We hypothesized that plastic and/or evolved changes in mitochondrial physiology help improve energy supply and mitigate ROS damage at high altitude.

## MATERIALS AND METHODS

### Animals and environmental exposures

Adult deer mice, *Peromyscus maniculatus* (J. A. Wagner 1845), were caught at high altitude on the summit of Mount Blue Sky (formerly Mount Evans) in Colorado, USA (39°35′18″N, 105°38′38″W; ∼4350 m above sea level), and at low altitude in the rural areas surrounding Kearney, NB, USA (40°42′5.4″N, 99°04′58.7″W; ∼660 m above sea level). Mice were then transported to McMaster University (90 m above sea level; Hamilton, ON, Canada), where they were bred in captivity to produce multiple generations of lab-bred offspring from several distinct highland and lowland families. Most experiments were conducted on third-generation lab-raised offspring, except for the measurements of *in vivo* ROS production with MitoB (see below), which were performed on first-generation offspring because third-generation animals were not available at the time this experiment was conducted. These mice were raised to adulthood (6–14 months of age) at ∼25°C and a 12 h:12 h light:dark photoperiod, and were provided with unlimited access to water and standard rodent chow (Teklad Global 2018, 18% Protein Rodent Diet, Inotiv, West Lafayette, IN, USA). Adults from each population were then randomly assigned to one of two acclimation groups: control conditions of warm (23–25°C) normobaric normoxia (standard lab conditions); or cold (5°C) hypobaric hypoxia (partial pressure of O_2_ ∼12 kPa, barometric pressure ∼60 kPa; simulating pressure at ∼4300 m elevation) for 6–8 weeks. Hypobaria was maintained using specially designed chambers that have been described previously (Lui et al., 2015; Lau et al., 2017; Mahalingam et al., 2017), and were held in a room controlled at 5°C. Mice in hypobaric chambers were removed 2–3 times per week for <20 min for cage cleaning. After acclimation, mice were rapidly euthanized by inhalation of >5% isoflurane vapour (<1 min) followed immediately by cervical dislocation. Body mass was immediately measured, and the diaphragm, gluteus maximus, vastus medialis and vastus lateralis muscles were dissected and weighed. A portion of the diaphragm as well as the left hindlimb muscles were used for mitochondrial respirometry, and the remaining diaphragm portion and right vastus medialis were prepared for TEM. All procedures were approved by the McMaster University Animal Research Ethics Board in accordance with guidelines established by the Canadian Council on Animal Care.

### Mitochondrial physiology of permeabilized muscle fibres

Mitochondrial physiology was measured in permeabilized muscle fibres at 37°C using high-resolution respirometry and fluorometry (Oxygraph-2k with O2K-Fluorescence Module; Oroboros Instruments, Innsbruck, Austria). Immediately after dissection, muscle samples were placed in BIOPS buffer (2.77 mmol l^−1^ CaK_2_EGTA, 7.23 mmol l^−1^ K_2_EGTA, 5.77 mmol l^−1^ Na_2_ATP, 6.56 mmol l^−1^ MgCl_2_, 20 mmol l^−1^ taurine, 15 mmol l^−1^ sodium phosphocreatine, 20 mmol l^−1^ imidazole, 0.5 mmol l^−1^ dithiothreitol, 50 mmol l^−1^ potassium methane sulfonate, pH 7.1) and kept on ice. The fibres were gently separated with dissecting probes and tweezers under a stereomicroscope. The fibres were then transferred to BIOPS buffer containing 50 μg ml^−1^ saponin and incubated for 30 min to chemically permeabilize the fibres. The permeabilized fibres were then rinsed three times for 10 min in MIR05 respiration solution (0.5 mmol l^−1^ EGTA, 3 mmol l^−1^ MgCl_2_, 60 mmol l^−1^ potassium lactobionate, 20 mmol l^−1^ taurine, 10 mmol l^−1^ KH_2_PO_4_, 20 mmol l^−1^ Hepes, 110 mmol l^−1^ sucrose and 1 g ml^−1^ of fatty acid-free bovine serum albumin; pH 7.1) to rinse out endogenous metabolites. The fibres were then weighed by first removing them from BIOPS buffer and then absorbing the liquid surrounding the fibres using blotting paper, after which the fibres were immediately used for measurements.

Measurements of permeabilized fibres (1.5–4 mg wet mass) were made in 2 ml of MIR05 solution within respirometry chambers of the Oxygraph-2k. To ensure that respiration was not limited by the rate of O_2_ diffusion into the permeabilized fibres, the MIR05 solution was hyper-oxygenated to an O_2_ concentration ∼450 μmol l^−1^ by bubbling O_2_ into the chambers before each set of measurements. Hyper-oxygenation was repeated if necessary whenever O_2_ concentration approached ∼200 μmol l^−1^, which is sufficient to avoid diffusion limitation based on our past experience and established recommendations (Pesta and Gnaiger, 2012). Mitochondrial respiration was measured as the rate of O_2_ consumption and is expressed relative to tissue wet mass (nmol O_2_ min^−1^ mg^−1^). The rate of ROS emission was measured by fluorescence detection of resorufin, which is produced from the reaction of hydrogen peroxide (H_2_O_2_) and Amplex Ultra Red (Thermo Fisher Scientific, Toronto, ON, Canada) (Krumschnabel et al., 2015). This was accomplished by exogenous addition of 10 μmol l^−1^ Amplex Ultra Red, 5 U ml^−1^ superoxide dismutase (SOD), 1 U ml^−1^ horseradish peroxidase (HPR) and 15 μmol l^−1^ diethylenetriaminepentaacetic acid (DTPA). Exogenous H_2_O_2_ was used to calibrate the resorufin fluorescence signal immediately before the start of measurements.

Mitochondrial respiration and ROS emission were then measured throughout a substrate titration protocol, with sequential additions of pyruvate (5 mmol l^−1^) and malate (2 mmol l^−1^) to initiate leak respiration (*L*_N,PM_), ADP (5 mmol l^−1^) to stimulate oxidative phosphorylation (OXPHOS) (*P*_PM_), glutamate (10 mmol l^−1^) to maximally stimulate Complex I (*P*_PMG_), and succinate (20 mmol l^−1^) to maximally stimulate Complexes I and II (*P*_PMGS_). The chambers were then reoxygenated and cytochrome *c* (10 μmol l^−1^) was added as a quality control to assess the integrity of the outer mitochondrial membrane, which never increased respiration by more than 10%. Carbonyl cyanide *m*-chlorophenyl hydrazone (CCCP) was then slowly titrated (to ∼1 μmol l^−1^) to maximally uncouple the mitochondria and determine the capacity for electron transport (*E*) via complexes I and II (*E*_PMGS_). Finally, ascorbate (2 mmol l^−1^) followed by *N*,*N*,*N*′,*N*′,-tetramethyl-*p*-phenylenediamine (TMPD; 0.5 mmol l^−1^) was used to maximally stimulate complex IV (*P*_Tm_), after which sodium azide (10 mmol l^−1^) was added to inhibit complex IV and measure background TMPD oxidation (complex IV capacity was calculated as the difference in respiration before minus after the addition of azide). ROS emission rate is not reported after the addition of succinate, because cytochrome *c*, ascorbate and TMPD are strongly redox active. Respiration and ROS emission rates were measured for at least 3 min in each state to obtain steady values and are expressed relative to tissue wet mass.

### Transmission electron microscopy

The diaphragm and vastus medialis were fixed at resting muscle length as follows. The muscle sample was placed in 0.9% NaCl to maintain osmotic balance and preserve tissue integrity during handling on a dissection dish, and one end of the muscle was gently tied to a wooden toothpick using silk suture. The muscle was lightly stretched for a few seconds and allowed it to return to resting length, then the opposite end of the muscle was tied to the toothpick (the time and amount of stretch were roughly consistent across preparations). The toothpick and tissue were then submerged in fixative (2% glutaraldehyde in 0.1 mol l^−1^ sodium cacodylate buffer at pH 7.4) and fixed for 24 h at 4°C. Fixed samples were trimmed into smaller pieces and transferred to clean microcentrifuge tubes containing fresh fixative. These samples were then sent to the Canadian Centre for Electron Microscopy (CCEM) at McMaster University (Hamilton, ON, Canada), where they underwent post-fixation, dehydration, embedding, sectioning and imaging using standard protocols for TEM. Images acquired at 2000× magnification were used for quantification.

We made unbiased measurements of mitochondrial volume density using stereological methods that have been previously described (Egginton, 1990; Scott et al., 2009; Mahalingam et al., 2017). Mitochondria were classified as subsarcolemmal mitochondria if they were located directly adjacent to the cell membrane outside the myofibrils, and were classified as intermyofibrillar mitochondria if they were surrounded by myofibrils within the interior of the fibre. Image locations were selected randomly across the muscle section such that a representative distribution of fibre types exhibited by the individual were analysed. A sufficient number of images were analysed to account for heterogeneity (16 images per muscle per mouse), based on preliminary assessments of the number of images required to achieve a stable mean value for the muscle. All images were analysed at the same magnification by a single observer who was blinded to sample identity throughout image analysis. Sample identity was revealed only after all measurements had been completed.

We also performed an exploratory quantification of oxidative fibre density and capillarity in the images used for quantifying mitochondrial volume. Muscle fibres exhibited large and characteristic differences in mitochondrial volume density that were used to visually classify them as either oxidative (high mitochondrial volume density) or glycolytic (low mitochondrial volume density), and the numerical density of oxidative fibres was calculated as the number of oxidative fibres divided by the total number of fibres. Fibre classification was performed consistently across all samples. Capillaries were also identified visually, and capillarity was quantified and reported here as capillary density (the number of capillaries per unit area) and capillary-to-fibre ratio.

### *In vivo* ROS production using MitoB

Mitochondrial H_2_O_2_ steady-state concentration was measured *in vivo* using the MitoB probe. When injected into the animal, the MitoB probe accumulates in mitochondria and is converted to MitoP in the presence of H_2_O_2_ (Cochemé et al., 2012), such that the ratio of MitoP to MitoB is proportional to mitochondrial H_2_O_2_ concentration (Cochemé et al., 2012; Salin et al., 2018; Lau et al., 2019; Ducros et al., 2024; Saleem et al., 2025). Mice received an intraperitoneal injection of a standard dose of MitoB (150 μl of 167 μmol l^−1^ MitoB; 25 nmol per mouse) (17116, Cayman Chemical, Ann Arbor, MI, USA), and were then returned to their cages in the appropriate acclimation condition and allowed to resume normal activity. After 6 h, mice were euthanized via isoflurane overdose followed by cervical dislocation, and each muscle was dissected, flash-frozen in liquid nitrogen and stored at −80°C for later analysis. The subsequent extraction of MitoB and MitoP (using a commercially available kit from Cayman Chemical) and quantification using LC-MS/MS were carried out as previously described in detail (Saleem et al., 2025). The mice used for these measurements were also used to measure mitochondrial H_2_O_2_ generation in the heart as part of another study (Saleem et al., 2025), but skeletal muscle measurements have not been previously reported.

### Statistics

Statistical analyses were performed in R version 4.4.0 (https://www.r-project.org/) using standard linear models. We tested for the effects of population, environment, population×environment and sex for measurements of mitochondrial respiration, ROS emission and substrate control ratio in each respiration state, each TEM measurement and the MitoP/MitoB ratios in each muscle. We used the same approach for muscle mass, but also included generation as an additional fixed effect. We used the lmTest package (Kuznetsova et al., 2017) to generate ANOVA *F*- and *P*-values. The full ANOVA results can be found in Tables S1 and S3–S6. The fixed effects of sex were generally not significant (except for capillary density in vastus medialis, *P*=0.043), so are not described further. When effects from ANOVA were significant, Holm–Šidák *post hoc* analysis was performed using the emmeans package (version 1.10.3; https://CRAN.R-project.org/package=emmeans) to make pairwise comparisons between populations within each environment and/or between environments within each population. Data are generally reported as means±s.e.m., and individual values are also reported for data presented in figures. *P*<0.05 was considered significant.

## RESULTS

### Muscle mass

Acclimation environment affected the mass of some muscles in one or both populations (Table 1; Table S1). Diaphragm mass increased in response to cold hypoxia (environment effect, *P*<0.001) by ∼15% in low-altitude mice and ∼24% in high-altitude mice. The acclimation response of the gluteus maximus differed between populations (population×environment, *P*<0.001), as mass increased ∼55% in cold hypoxia in high-altitude mice, whereas cold hypoxia did not affect mass in low-altitude mice. In contrast, body mass and the mass of the vastus medialis and vastus lateralis did not vary across populations or environments.

**Table 1.** Body and muscle mass from high-altitude and low-altitude populations of deer mice acclimated to warm normoxia or cold hypoxia

	Low-altitude mice		High-altitude mice		
	Warm normoxia	Cold hypoxia	Warm normoxia	Cold hypoxia	Significant effects
*N*	21	29	21	26	
Body mass (g)	23.0±1.2	22.1±0.7	20.9±0.6	20.3±0.6	NS
Diaphragm mass (mg g^−1^)	4.48±0.18	5.15±0.23**	4.52±0.22	5.60±0.24**	Env
Gluteus maximus mass (mg g^−1^)	4.84±0.37	4.64±0.20	4.40±0.33	6.84±0.53*,**	Pop; Env; Pop×Env
Vastus medialis mass (mg g^−1^)	0.947±0.070	0.974±0.049	0.970±0.050	1.08±0.07	NS
Vastus lateralis mass (mg g^−1^)	2.83±0.16	2.87±0.10	3.09±0.14	2.99±0.14	NS

Body mass (g) and muscle mass (mg per g body mass) are presented as means±s.e.m. Significant effects from ANOVA for population (Pop), acclimation environment (Env) or the Pop×Env interaction are summarized, and full ANOVA results are provided in Table S1. NS, not significant. *Significant (*P*<0.05) pairwise differences between highlanders and lowlanders within the same acclimation environment; **significant (*P*<0.05) pairwise differences between acclimation environments within a population in Holm–Šidák *post hoc* tests.

### Variation in mitochondrial respiratory capacity of permeabilized muscle fibres

**Fig. 1.**
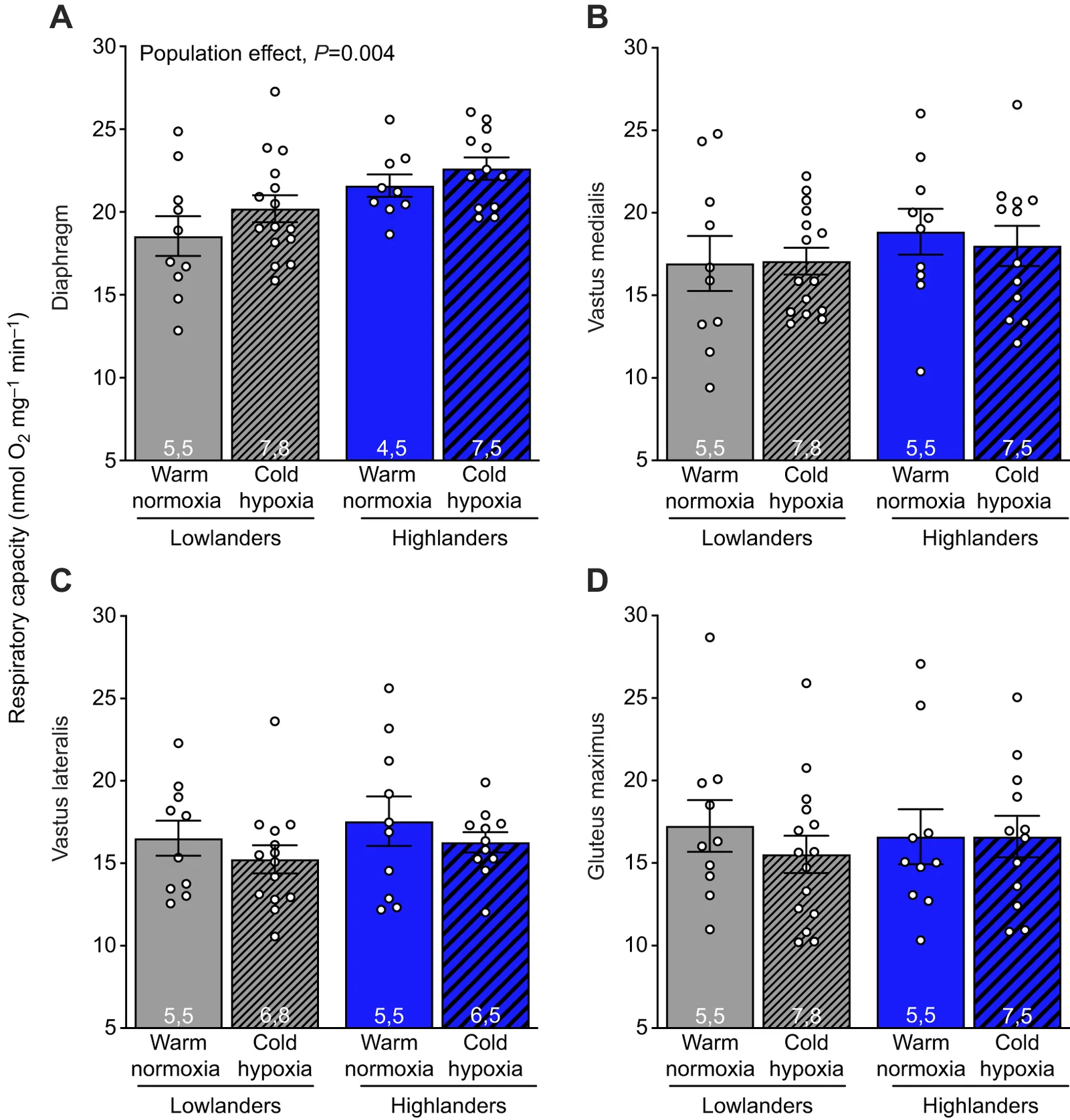
**Mitochondrial respiratory capacity was increased in the diaphragm in high-altitude mice.** Mitochondrial respiratory capacity was measured in permeabilized muscle fibres from the diaphragm (A), vastus medialis (B), vastus lateralis (C) and gluteus maximus (D) of high-altitude and low-altitude populations of deer mice (‘highlanders’ and ‘lowlanders’) acclimated to warm normoxia (25°C, ∼20 kPa O_2_) or cold hypoxia (5°C, ∼12 kPa O_2_) for 6–8 weeks. Maximal oxidative phosphorylation (OXPHOS) respiration was stimulated with ADP and substrates of complexes I and II (pyruvate, malate, glutamate and succinate). Data are shown with circles representing values for individual mice and bars representing means±s.e.m.; the number of males and females in each group is indicated within the bars (*N*_males_, *N*_females_). Significant ANOVA *P*-values for fixed effects are shown in each panel, and full ANOVA results from linear models are provided in Tables S3–S6. Respiration data collected for the full substrate titration protocol can be found in Table S2.

The variation in mitochondrial respiratory function between populations and environments differed between muscles. Respiration measured in phosphorylating conditions with substrates of complexes I and II (*P*_PMGS_) was used to reflect each muscle's capacity for mitochondrial OXPHOS. In the diaphragm, mitochondrial OXPHOS capacity differed between populations (*P*=0.004), with ∼13.5% greater capacity in high-altitude mice than in low-altitude mice (Fig. 1A). Similar population differences were exhibited in phosphorylating conditions with only substrates of complex I: pyruvate and malate (*P*_PM_, *P*=0.003); and pyruvate, malate and glutamate (*P*_PMG_, *P*=0.012) (Tables S2 and S3). There was also a significant environment effect on *P*_PM_ (*P*=0.041), driven by increases of ∼11–13% in response to cold hypoxia in both populations (Tables S2 and S3). In contrast, the effects of neither population (*P*≥0.204) nor environment (*P*≥0.223) on OXPHOS capacity were significant in the other muscles (Fig. 1B–D; Tables S2 and S4–S6).

**Fig. 2.**
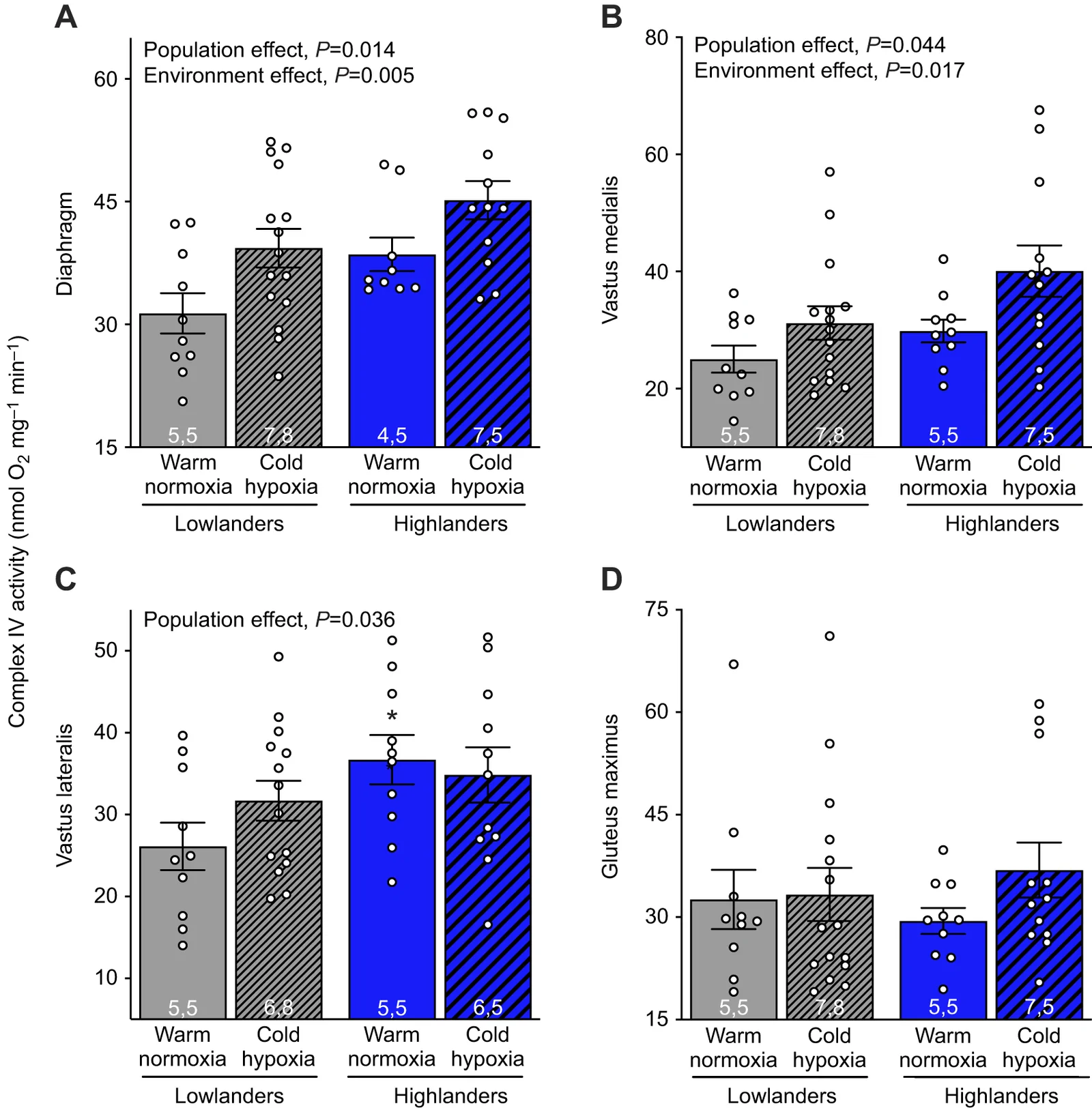
**The respiratory capacity of complex IV increased in cold hypoxia and was greater in high-altitude mice in several muscles.** Complex IV respiration was measured in permeabilized muscle fibres from the diaphragm (A), vastus medialis (B), vastus lateralis (C) and gluteus maximus (D) of high-altitude and low-altitude populations of deer mice (‘highlanders’ and ‘lowlanders’) acclimated to warm normoxia (25°C, ∼20 kPa O_2_) or cold hypoxia (5°C, ∼12 kPa O_2_) for 6–8 weeks. Complex IV respiration was stimulated with ascorbate and TMPD, and subsequent addition of sodium azide was used to inhibit complex IV to account for background TMPD oxidation. Data are shown with circles representing values for individual mice and bars representing means±s.e.m.; the number of males and females in each group is indicated within the bars (*N*_males_, *N*_females_). Significant ANOVA *P*-values for fixed effects are shown in each panel. The asterisk indicates a significant (*P<0.05) pairwise difference between highlanders and lowlanders within an acclimation environment.

The variation in maximal respiration via complex IV (cytochrome *c* oxidase) also differed between muscles, but was generally more pronounced than the variation in OXPHOS capacity (Fig. 2). In the diaphragm, complex IV respiration (*P*_Tm_, stimulated by ascorbate and TMPD) increased by ∼17–25% in response to cold hypoxia in both populations (environment effect, *P=*0.005) and was ∼17% greater overall in high-altitude mice compared with low-altitude mice (population effect, *P*=0.014) (Fig. 2A; Table S3). A similar pattern of variation was observed in the vastus medialis, in which complex IV respiration increased in response to cold hypoxia (environment effect, *P=*0.017) and was greater overall in high-altitude mice (population effect, *P*=0.044) (Fig. 2B; Table S4). Complex IV respiration was also greater in high-altitude mice in the vastus lateralis (population effect, *P*=0.036) but there was no environment effect in this muscle (*P*=0.487) (Fig. 2C; Table S5). Only the gluteus maximus showed no significant effects of population (*P*=0.577) or environment (*P*=0.775) on complex IV respiration (Fig. 2D; Table S6).

We next calculated substrate control ratios to further examine mitochondrial flux control (Table 2). These ratios provide insight into potential variation in the relative influence of different substrates on overall OXPHOS capacity. The diaphragm exhibited no variation in any substrate control ratios (*P*_PM_/*P*_PMGS_, *P*_PMG_/*P*_PMGS_) nor was there any variation in the excess capacity of the electron transport system relative to OXPHOS (*E*_PMGS_/*P*_PMGS_) (Table 2; Table S3), suggesting that there were no changes in mitochondrial flux control in association with the population differences in OXPHOS capacity in this muscle (Fig. 1). Similarly, there was no significant variation in these ratios in the vastus medialis or vastus lateralis (Table 2; Tables S4 and S5). In the gluteus maximus, however, *P*_PM_/*P*_PMGS_ was ∼1.2-fold greater in high-altitude mice compared with low-altitude mice (population effect, *P*=0.044) and increased by ∼1.1-fold in response to cold hypoxia in both populations (environment effect, *P*=0.005) (Table 2; Table S6), reflecting an increased relative capacity for pyruvate oxidation. In addition, *E*_PMGS_/*P*_PMGS_ increased ∼1.1-fold in cold hypoxia in both populations (Table 2; Table S6), which could indicate an increased influence of the phosphorylation system over OXPHOS.

**Table 2.** Substrate control ratios for mitochondrial respiration from high-altitude and low-altitude populations of deer mice acclimated to warm normoxia or cold hypoxia

	Low-altitude mice		High-altitude mice		
	Warm normoxia	Cold hypoxia	Warm normoxia	Cold hypoxia	Significant effects
Diaphragm					
* P*_PM_/*P*_PMGS_	0.875±0.059 (10)	0.889±0.048 (15)	0.875±0.025 (9)	0.927±0.033 (12)	NS
* P*_PMG_/*P*_PMGS_	0.944±0.033 (10)	0.917±0.014 (15)	0.915±0.013 (9)	0.917±0.020 (12)	NS
* E*_PMGS_/*P*_PMGS_	1.25±0.04 (10)	1.14±0.04 (15)	1.11±0.06 (9)	1.16±0.04 (12)	NS
Vastus medialis					
* P*_PM_/*P*_PMGS_	0.791±0.044 (10)	0.793±0.027 (15)	0.766±0.022 (10)	0.834±0.036 (12)	NS
* P*_PMG_/*P*_PMGS_	0.910±0.017 (10)	0.862±0.019 (15)	0.846±0.033 (10)	0.864±0.021 (12)	NS
* E*_PMGS_/*P*_PMGS_	1.15±0.03 (10)	1.13±0.02 (15)	1.05±0.03 (10)	1.12±0.05 (12)	NS
Vastus lateralis					
* P*_PM_/*P*_PMGS_	0.812±0.042 (10)	0.817±0.029 (14)	0.769±0.050 (10)	0.882±0.025 (11)	NS
* P*_PMG_/*P*_PMGS_	0.927±0.020 (10)	0.889±0.017 (14)	0.829±0.047 (10)	0.878±0.029 (11)	NS
* E*_PMGS_/*P*_PMGS_	1.14±0.037 (10)	1.17±0.04 (14)	1.18±0.05 (10)	1.14±0.03 (11)	NS
Gluteus maximus					
* P*_PM_/*P*_PMGS_	0.737±0.036 (10)	0.828±0.034 (15)	0.802±0.032 (10)	0.906±0.026 (12)	Pop; Env
* P*_PMG_/*P*_PMGS_	0.891±0.020 (10)	0.904±0.016 (15)	0.909±0.023 (10)	0.934±0.013 (12)	NS
* E*_PMGS_/*P*_PMGS_	1.09±0.02 (10)	1.20±0.04 (15)	1.11±0.04 (10)	1.21±0.056 (12)	Env

Data are reported as means±s.e.m. Substrate control ratios for pyruvate oxidation (*P*_PM_) and maximal complex I respiration (*P*_PMG_) were calculated relative to maximal OXPHOS respiration (*P*_PMGS_). Maximal capacity for electron transport (*E*_PMGS_) was induced by uncoupling oxidative phosphorylation (OXPHOS) with carbonyl cyanide *m*-chlorophenyl hydrazone (CCCP). Subscripts represent the following substrates that were used to elicit each respiratory state: P, pyruvate; M, malate; G, glutamate; S, succinate. Significant effects from ANOVA for population (Pop), acclimation environment (Env) or the Pop×Env interaction are summarized, and full ANOVA results are provided in Tables S3–S6. NS, not significant.

### Expansion of mitochondrial volume density in cold hypoxia

**Fig. 3.**
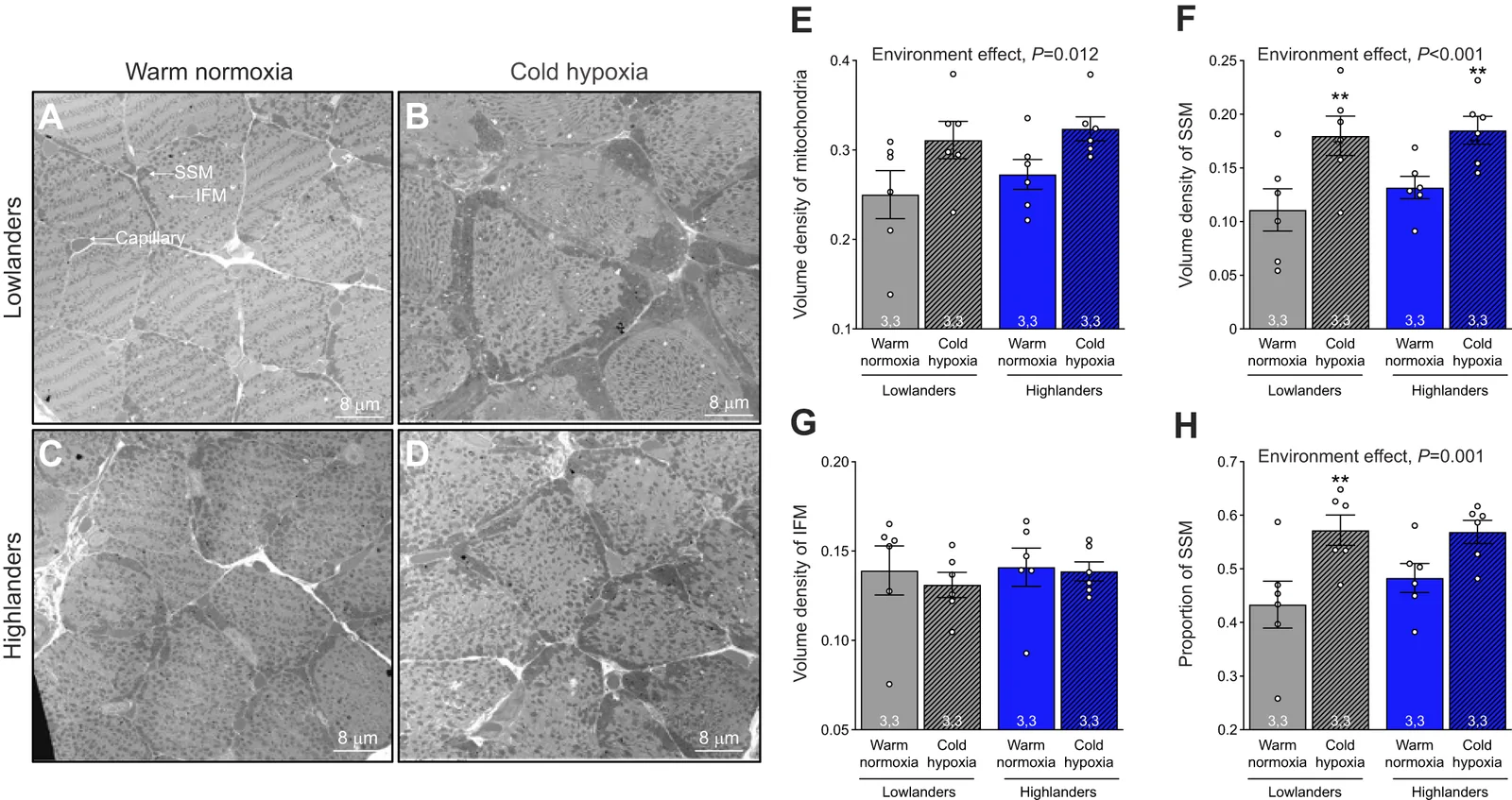
**Acclimation to cold hypoxia increased mitochondrial volume density in the diaphragm.** Representative transmission electron micrographs of low-altitude mice (‘lowlanders’) (A,B) and high-altitude mice (‘highlanders’) (C,D) acclimated to warm normoxia (25°C, ∼20 kPa O_2_) (A,C) or cold hypoxia (5°C, ∼12 kPa O_2_) (B,D) for 6–8 weeks. Stereological methods were used to quantify the volume density of all mitochondria (E), subsarcolemmal mitochondria (SSM) (F) and intermyofibrillar mitochondria (IFM) (G), as well as the proportion of SSM relative to total mitochondria (H). Data are shown with circles representing values for individual mice and bars representing means±s.e.m.; the number of males and females in each group is indicated within the bars (*N*_males_, *N*_females_). Significant ANOVA *P*-values for fixed effects are shown in each panel, and full ANOVA results from linear models are provided in Table S3. Holm–Šidák *post hoc* tests were performed when there were significant fixed-effects in ANOVA: **Significant (*P*<0.05) pairwise differences between acclimation environments within a population.

**Fig. 4.**
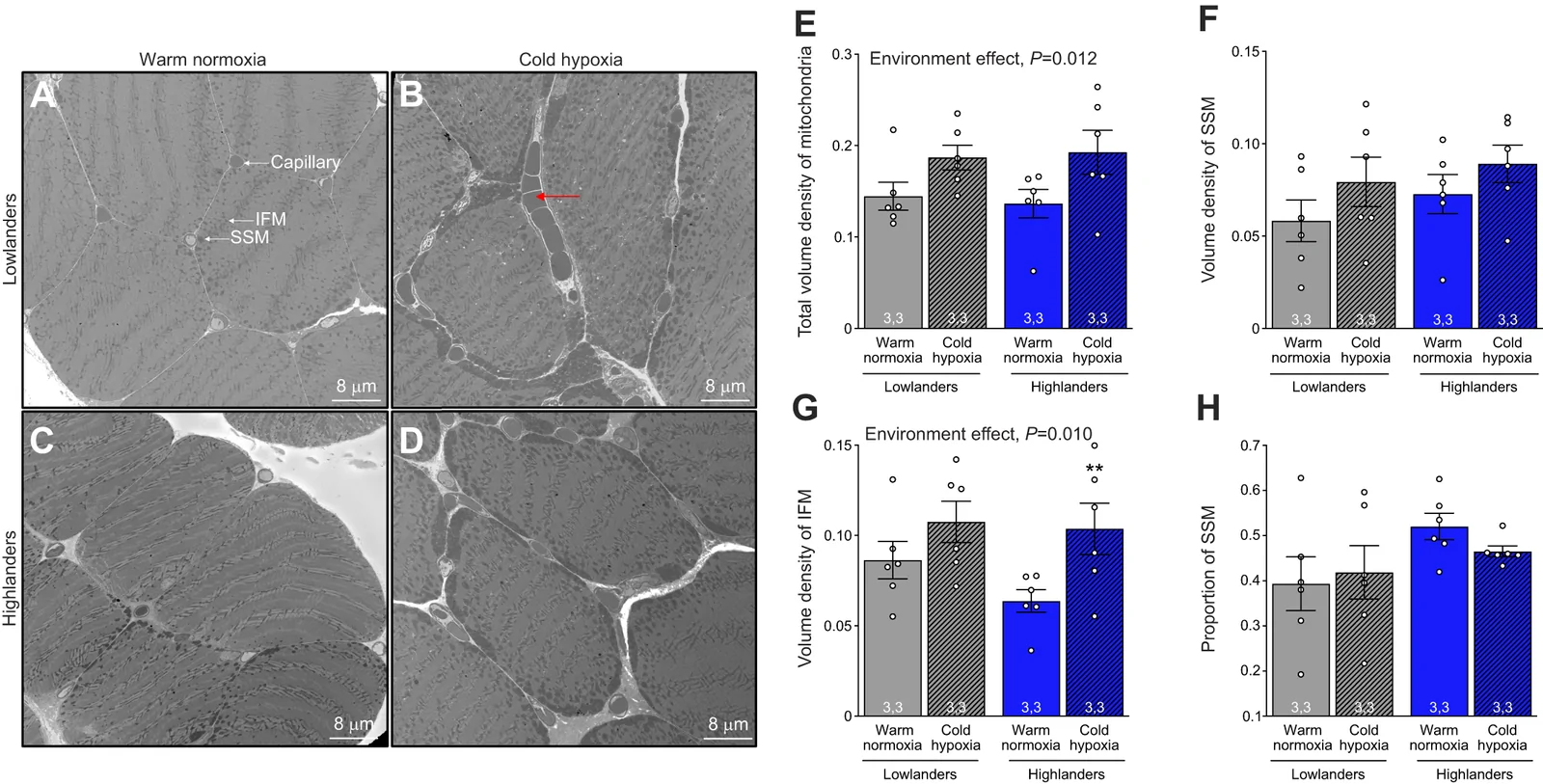
**Acclimation to cold hypoxia increased mitochondrial volume density in the vastus medialis.** Representative transmission electron micrographs of low-altitude mice (‘lowlanders’) (A,B) and high-altitude mice (‘highlanders’) (C,D) acclimated to warm normoxia (25°C, ∼20 kPa O_2_) (A,C) or cold hypoxia (5°C, ∼12 kPa O_2_) (B,D) for 6–8 weeks. Many capillaries were observed in transverse section in TEM images, reflecting a roughly parallel orientation to the muscle fibres (white labelled arrow), but there were some tortuous capillaries oriented at perpendicular or oblique angles relative to the muscle fibres (red arrow). Stereological methods were used to quantify the volume density of all mitochondria (E), subsarcolemmal mitochondria (SSM) (F) and intermyofibrillar mitochondria (IFM) (G), and the proportion of SSM relative to total mitochondria was also calculated (H). Data are shown with circles representing values for individual mice and bars representing means±s.e.m.; the number of males and females in each group is indicated within the bars (*N*_males_, *N*_females_). Significant ANOVA *P*-values for fixed effects are shown in each panel, and full ANOVA results from linear models are provided in Table S4. Holm–Šidák *post hoc* tests were performed when there were significant fixed effects in ANOVA: **Significant (*P*<0.05) pairwise differences between acclimation environments within a population.

Mitochondrial volume density increased across muscles in cold hypoxia (Figs 3 and 4; Tables S3 and S4). TEM was used to measure mitochondrial volume density in the diaphragm and vastus medialis, for which representative images are shown in Figs 3A–D and 4A–D. In the diaphragm, total mitochondrial volume density increased by ∼19–24% in response to cold hypoxia (environment effect, *P*=0.012) but there were no differences between populations (population effect, *P*=0.393; population×environment, *P*=0.808) (Fig. 3). The effect of acclimation environment was driven by an increased abundance of subsarcolemmal mitochondria, whose volume density increased by ∼41–62% in cold hypoxia (environment effect, *P*<0.01), with no variation in the volume density of intermyofibrillar mitochondria. As a result, the proportion of subsarcolemmal mitochondria relative to total mitochondrial volume also increased in cold hypoxia (environment effect, *P*=0.001). Similar patterns of variation were observed in the vastus medialis for total mitochondrial volume density, which increased by ∼29–41% in response to cold hypoxia in both populations (environment effect, *P*=0.012), with no differences between populations (population effect, *P*=0.951; population×environment, *P*=0.700) (Fig. 4). However, in this muscle, cold hypoxia increased intermyofibrillar mitochondria volume density by ∼25–63% (environment effect, *P*<0.01). There was a similar pattern of variation in subsarcolemmal mitochondria volume density, although the effects of cold hypoxia did not reach significance (environment effect, *P=*0.125), and the proportion of subsarcolemmal mitochondria relative to total mitochondrial volume did not change.

TEM images (Figs 3 and 4) were also used to quantify capillarity and muscle fibre composition in the diaphragm and vastus medialis (Table 3). In the diaphragm, the ratio of capillaries to muscle fibres increased by ∼17.1% and ∼29.5% in response to cold hypoxia in low- and high-altitude mice, respectively (environment effect, *P*<0.001), with no significant population effect or population×environment interaction (Table 3; Table S3). However, capillary density was reduced in response to cold hypoxia, decreasing by ∼13.9% and ∼17.3% in lowland and highland mice, respectively (environment effect, *P*=0.015), possibly due to an unquantified increase in muscle fibre size (Table 3; Table S3). In the vastus medialis, however, both capillary density and capillary-to-fibre ratio increased in response to cold hypoxia (environment effects, *P*=0.022 and 0.023), with no significant differences between populations (Table 3; Table S4). The numerical density of oxidative fibres did not vary between environments or populations in either muscle (Table 3; Tables S3 and S5).

**Table 3.** Capillarity and oxidative fibre density in diaphragm and vastus medialis from high-altitude and low-altitude populations of deer mice acclimated to warm normoxia or cold hypoxia.

	Low-altitude mice		High-altitude mice		
	Warm normoxia	Cold hypoxia	Warm normoxia	Cold hypoxia	Significant effects
Diaphragm					
* *Capillary density (mm^−2^)	2302.5±186.3 (6)	1982.5±103.0 (6)	2203.8±124.8 (6)	1821.7±86.6 (6)	Env
* *Capillary to fibre ratio	1.01±0.05 (6)	1.18±0.05** (6)	0.935±0.027 (6)	1.21±0.05** (6)	Env
* *Numerical density of oxidative fibres	0.945±0.033 (6)	0.952±0.031 (6)	0.945±0.028 (6)	0.888±0.026 (6)	NS
Vastus medialis					
* *Capillary density (mm^−2^)	1280.9±132.5 (6)	1600.6±76.1 (6)	1433.6±115.9 (6)	1616.9±105.9 (6)	Env
* *Capillary to fibre ratio	1.29±0.15 (6)	1.64±0.08 (6)	1.38±0.14 (6)	1.636±0.091 (6)	Env
* *Numerical density of oxidative fibres	0.513±0.080 (6)	0.739±0.069 (6)	0.645±0.088 (6)	0.700±0.060 (6)	NS

Data are reported as means±s.e.m. Significant effects from ANOVA for population (Pop), acclimation environment (Env) or the Pop×Env interaction are summarized, and full ANOVA results are provided in Tables S3 and S4. NS, not significant. **Significant (*P*<0.05) pairwise differences between environments within a population in Holm–Šidák *post hoc* tests.

### Cold hypoxia reduced mitochondrial ROS generation across skeletal muscles

**Fig. 5.**
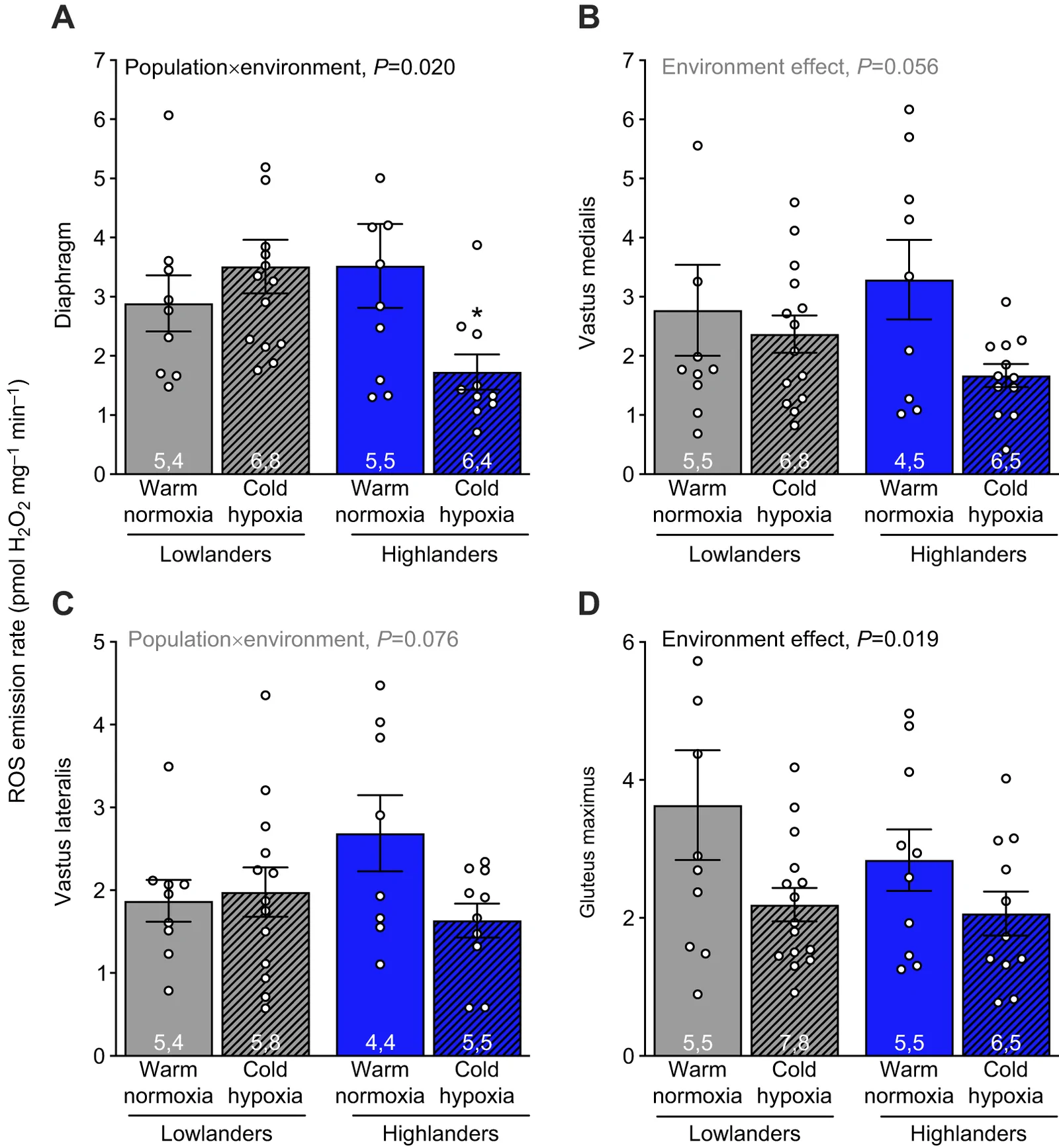
***Ex vivo* rates of mitochondrial reactive oxygen species (ROS) emission decreased in cold hypoxia, primarily in high-altitude mice.** Mitochondrial ROS emission was measured in permeabilized skeletal muscle from the diaphragm (A), vastus medialis (B), vastus lateralis (C) and gluteus maximus (D) of high-altitude and low-altitude populations of deer mice (‘highlanders’ and ‘lowlanders’) acclimated to warm normoxia (25°C, ∼20 kPa O_2_) or cold hypoxia (5°C, ∼12 kPa O_2_) for 6–8 weeks. ROS was measured by fluorescence detection of resorufin using Amplex Ultra Red (see Materials and Methods) during OXPHOS via complexes I and II, concurrent with the respiration data shown in Fig. 1. Data are presented with circles representing values for individual mice, except for a few individual values that are were beyond the *y-*axis limits (diaphragm, cold hypoxic lowlander at 8.11 pmol H_2_O_2_ mg^−1^ min^−1^ and warm normoxic highlander at 8.74 pmol H_2_O_2_ mg^−1^ min^−1^; vastus medialis, warm normoxic lowlander at 8.46 pmol H_2_O_2_ mg^−1^ min^−1^; gluteus maximus, warm normoxic lowlander at 9.13 pmol H_2_O_2_ mg^−1^ min^−1^) and bars representing means±s.e.m.; the number of males and females in each group is indicated within the bars (*N*_males_, *N*_females_). Significant ANOVA *P*-values for fixed effects are shown in each panel, and full ANOVA results from linear models are provided in Tables S3–S6. Holm–Šidák *post hoc* tests were performed when there were significant fixed effects in ANOVA: *Significant (*P*<0.05) pairwise differences between highlanders and lowlanders within an acclimation environment. ROS emission data collected for the full substrate titration protocol can be found in Table S2.

Rates of mitochondrial ROS emission measured *ex vivo* were often reduced after acclimation to cold hypoxia across muscles, particularly in high-altitude mice (Fig. 5; Tables S2–S6). In the diaphragm, there was a pronounced population difference in the effects of cold hypoxia on mitochondrial ROS emission during OXPHOS via complexes I and II (*P*_PMGS_) (population×environment, *P*=0.020), driven by a ∼51% reduction after cold hypoxia acclimation in high-altitude mice but no change in low-altitude mice (Fig. 5A). The vastus medialis and vastus lateralis exhibited similar patterns of variation, in which high-altitude mice tended to reduce ROS emission during *P*_PMGS_ in cold hypoxia by a greater magnitude on average, but the effects were only marginally significant (environment effect in vastus medialis, *P*=0.056; population×environment in vastus lateralis, *P=*0.076). However, cold hypoxia significantly decreased mitochondrial ROS emission during OXPHOS via complex I (*P*_PMG_) in both muscles (environment effects, *P*=0.039 and 0.018, respectively), and these effects were driven by much larger decreases in high-altitude mice (Tables S2, S4 and S5). Acclimation to cold hypoxia also decreased ROS emission by ∼27–40% in the gluteus maximus (environment effect, *P*=0.019).

**Fig. 6.**
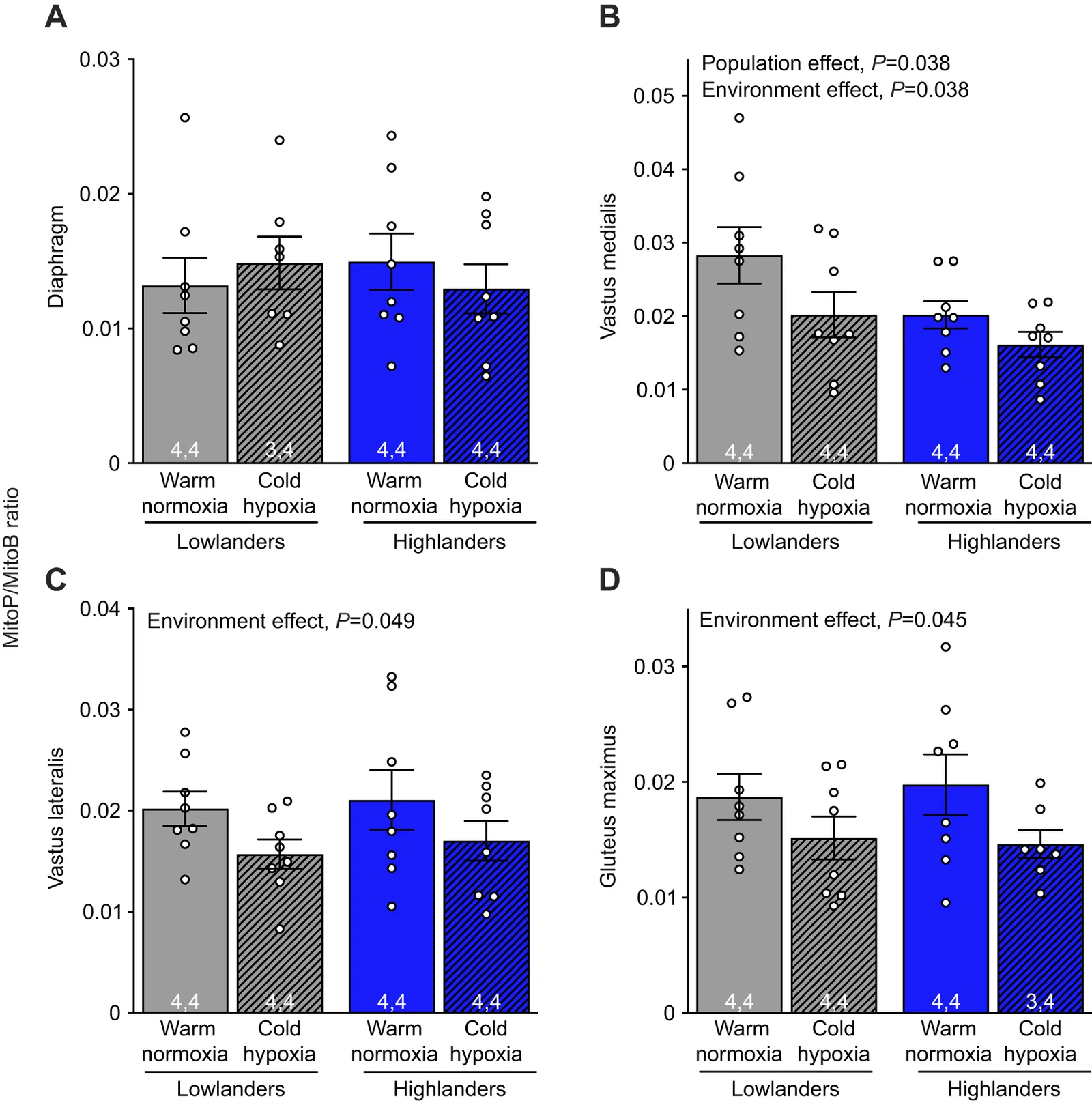
***In vivo* rates of mitochondrial ROS generation decreased after cold hypoxia acclimation across skeletal muscles.** The MitoP/MitoB ratio was measured as an index of mitochondrial H_2_O_2_ in the diaphragm (A), vastus medialis (B), vastus lateralis (C) and gluteus maximus (D) of high-altitude and low-altitude populations of deer mice (‘highlanders’ and ‘lowlanders’) acclimated to warm normoxia (25°C, ∼20 kPa O_2_) or cold hypoxia (5°C, 12 kPa O_2_) for 8 weeks. Circles represent values for individual mice and bars represent means±s.e.m.; the number of males and females in each group is indicated within each bar (*N*_males_, *N*_females_). Significant ANOVA *P*-values for fixed effects are shown in each panel, and the full ANOVA results from linear models are provided in Tables S3–S6.

The lower rates of mitochondrial ROS emission measured *ex vivo* were sometimes mirrored by similar variation in mitochondrial ROS generation measured *in vivo* using MitoB (Fig. 6; Tables S4–S6). Acclimation to cold hypoxia often decreased *in vivo* ROS generation, as reflected by significant main effects of the environment on MitoP/MitoB in the vastus medialis (*P*=0.038), vastus lateralis (*P*=0.049) and gluteus maximus (*P*=0.045). Furthermore, in the vastus medialis, high-altitude mice exhibited ∼25% lower MitoP/MitoB values than low-altitude mice (population effect, *P*=0.038) (Fig. 6B). However, in contrast to the variation in *ex vivo* measurements, the MitoP/MitoB values in the diaphragm did not vary between populations or acclimation environments (Fig. 6A).

## DISCUSSION

Our study shows that plasticity and evolved changes in mitochondrial physiology shape the phenotype of skeletal muscles in small mammals at high altitude. Acclimation to cold hypoxia increased mitochondrial abundance, respiration and/or complex IV activity (Figs 1–4) and decreased mitochondrial ROS generation (Figs 5 and 6) in one or more muscles. High-altitude mice had greater mitochondrial respiration in the diaphragm as well as greater complex IV activity in several muscles compared with low-altitude mice, and also exhibited greater decreases in mitochondrial ROS emission in response to cold hypoxia. Our findings suggest that plastic and evolved changes in mitochondrial physiology likely help improve energy supply and mitigate oxidative stress at high altitude.

The elevated mitochondrial OXPHOS capacity in the diaphragm of high-altitude deer mice (Fig. 1A) could help support the high demands of breathing in cold hypoxia. Total ventilation is greatly increased in cold hypoxia at high altitude, as a result of the combined effects of the hypoxic ventilatory response and the metabolic response to cold (Ivy and Scott, 2017; Tate et al., 2020). High-altitude deer mice also breathe with deeper tidal volumes but lower breathing frequencies compared with low-altitude mice, a breathing pattern that improves alveolar ventilation and thus enhances arterial oxygenation (Ivy and Scott, 2017, 2018). Although ventilatory measurements were not included here, the observed differences in mitochondrial respiratory capacity in the diaphragm should be advantageous for meeting the elevated energetic demands of breathing during cold hypoxia (Gamboa and Andrade, 2012; Lewis and Halloran, 2016). Our findings here are consistent with previous studies showing that cytochrome *c* oxidase activity is elevated in the diaphragm of high-altitude deer mice, although the current study is the first to examine the diaphragm of mice in cold hypoxia (Lui et al., 2015; Dawson et al., 2018; Garrett et al., 2024). The absence of any population differences in mitochondrial volume density (Fig. 3) suggests that high-altitude mice have enhanced OXPHOS capacity per mitochondrial volume, which could arise from evolved changes in expression levels or functional properties of cytochrome *c* oxidase and other OXPHOS complex proteins. Considered together, these findings highlight the diaphragm as a critical site of mitochondrial adaptations that likely underpin effective ventilation and oxygen uptake at high altitude.

The locomotory muscles examined in this study did not exhibit any differences in OXPHOS capacity (Fig. 1B–D). This observation differs from the gastrocnemius, in which high-altitude mice have greater OXPHOS capacity due to the higher volume density and OXPHOS capacity of subsarcolemmal mitochondria (Mahalingam et al., 2017, 2020; Dawson and Scott, 2022). Although all these muscles can be involved in both locomotion and shivering thermogenesis (Israel and Pozos, 1989; Hamner et al., 2010; Rowland et al., 2015), there could be differences in their relative use and energetic demands at high altitude. Different muscles vary in their overall activation and patterns of motor unit recruitment during changes in posture, locomotion or shivering (Franz and Kram, 2012; Lee et al., 2013). EMG measurements suggest that the gastrocnemius in particular tends to be active over a greater proportion of time than the other muscles during routine locomotion (Pearson et al., 2005; Akay et al., 2014). Furthermore, measurements of muscle fatty acid uptake suggest that the gastrocnemius has greater uptake rates than both the vastus lateralis and the gluteus maximus during cold challenge (Lyons and McClelland, 2024). If differences similarly exist between muscles in the use of shivering during cold challenge, this could lead to greater selective pressure at high altitude for enhanced mitochondrial respiratory capacity in the gastrocnemius relative to the other locomotory muscles examined here. These findings highlight the importance of examining multiple skeletal muscles with different functions and energetic requirements to obtain a comprehensive understanding of the role of skeletal muscles in environmental adaptation.

Complex IV activity was elevated in high-altitude mice across the diaphragm, vastus medialis and vastus lateralis (Fig. 2). It has been suggested that the excess capacity of complex IV relative to OXPHOS is advantageous for maintaining mitochondrial respiration at low O_2_ levels, because greater excess capacity reduces the relative catalytic activity of complex IV and increases mitochondrial O_2_ affinity (Gnaiger et al., 1998). Such an effect could be advantageous in high-altitude deer mice as a mitochondrial mechanism to help maintain muscle activity and thermogenesis in cold hypoxia. However, there have been few manipulative tests of the potential relationship between complex IV excess capacity and mitochondrial O_2_ affinity, but such tests would be a valuable subject of future research.

Chronic exposure to cold hypoxia increased mitochondria volume density in both the diaphragm (Fig. 3) and vastus medialis (Fig. 4), consistent with the effects of cold acclimation on mitochondrial volume density in other mammal species (Buser et al., 1982; Bal et al., 2017a,b). The increase in mitochondrial volume density in the diaphragm was driven entirely by an increased abundance of subsarcolemmal mitochondria (Fig. 3), the fraction of mitochondria found adjacent to the sarcolemma and nearest capillaries (Glancy et al., 2015; Parry et al., 2024; Schell et al., 2024). The preferential enrichment of subsarcolemmal mitochondria has been reported in numerous other high performing mammals and birds (Buser et al., 1982; Suarez et al., 1991; Herpin and Lefaucheur, 1992; Scott et al., 2009; Mahalingam et al., 2017; Schell et al., 2024), and may be advantageous for reducing diffusion distances between capillaries and mitochondria for oxygen and metabolic substrates. However, the increase in mitochondrial volume density in the vastus medialis was associated with an increased abundance of intermyofibrillar mitochondria (Fig. 4), which has been observed after chronic exposure to hypoxia in skeletal muscles of humans and rats (van Ekeren et al., 1992; Jacobs et al., 2016; Schytz et al., 2025). These acclimation-induced increases in mitochondrial abundance would be expected to increase the OXPHOS capacity of the tissue, but empirical support for this expectation was inconsistent. Cold hypoxia acclimation increased complex IV activity in both muscles (Fig. 2), increased OXPHOS respiration with pyruvate and malate in the diaphragm (Table S2), and increased average OXPHOS capacity in the diaphragm (Fig. 1), but cold hypoxia had no effect on OXPHOS capacity in the vastus medialis (Fig. 1). This mismatch suggests that the respiratory capacity per unit mitochondrial volume may have been lower after cold hypoxia acclimation, which could arise as a result of lower cristae density and/or membrane packing of electron transport system proteins (Cogliati et al., 2016; Heine et al., 2023).

Chronic exposure to cold hypoxia also reduced mitochondria ROS emission *in vivo* and/or *ex vivo* across muscles, particularly in high-altitude mice (Figs 5 and 6). Chronic exposure to hypoxia alone (i.e. not combined with cold) has been previously shown to reduce *ex vivo* mitochondrial ROS emission in deer mice (Mahalingam et al., 2017; Dawson and Scott, 2022) and several other hypoxia-tolerant species, including freshwater turtle, epaulette shark and killifish (Hickey et al., 2012; Du et al., 2016; Bundgaard et al., 2018). Mitochondrial ROS emission reflects the balance between ROS production by the electron transport system and ROS consumption by intra-mitochondrial antioxidants, so the reduction observed across muscles in this study could result from decreased electron leak and/or enhanced ROS scavenging (Munro and Treberg, 2017). These possibilities were not explored here, but recent research on cardiac mitochondria suggested that high-altitude deer mice have greater rates of mitochondrial ROS consumption via the thioredoxin-dependent pathway, which reduces mitochondrial ROS emission both *ex vivo* and *in vivo* (Saleem et al., 2025). Similarly, the reductions in *ex vivo* mitochondrial ROS emission in the gluteus maximus and vastus medialis after cold hypoxia acclimation were mirrored by reductions in mitochondrial ROS levels *in vivo*. In the diaphragm, however, the reduction in *ex vivo* mitochondrial ROS emission in high-altitude mice was not associated with comparable changes *in vivo*. This discrepancy may reflect the differences between *ex vivo* and *in vivo* conditions, because the former reflects intrinsic mitochondrial properties under standardized experimental conditions whereas the latter reflects mitochondrial ROS emission that is realized in the dynamic physiological conditions within the intact muscle. Indeed, *in vivo* mitochondrial ROS levels are context dependent and could be influenced by the metabolic activity required for ventilatory muscle contraction, associated variation in oxygen delivery and substrate utilization, and cytosolic redox state (Murphy, 2009; Brand, 2016; Munro and Treberg, 2017). It is also possible that captive breeding had diaphragm-specific effects on mitochondrial ROS emission, because different generations were used for measurements *in vivo* (1st) versus *ex vivo* (3rd). Nevertheless, the variation across muscles suggests that adjustments in mitochondrial ROS metabolism could be valuable for maintaining redox homeostasis and mitigating oxidative stress in the cold hypoxic environment at high altitude (Dosek et al., 2007; Saleem et al., 2025).

Overall, our results support growing evidence that plasticity and evolved changes in mitochondrial traits contribute to the regulation of energy and ROS metabolism at high altitude. Evidence across skeletal muscles suggest that increases in mitochondrial abundance, subcellular distribution, respiratory capacity and complex IV activity may help improve cellular energy supply in cold hypoxia. Reductions in mitochondrial ROS emission may help reduce oxidative stress and/or alter cellular ROS signalling. Therefore, adjustments to mitochondrial physiology in skeletal muscle may represent an important mechanism contributing to performance and adaptation at high altitude.

## Supplementary Material

10.1242/jexbio.252523_sup1 Supplementary information
