# Supplementary material for: Plasticity and evolved changes in mitochondrial physiology across skeletal muscles of high-altitude deer mice (Peromyscus maniculatus)
Source: J Exp Biol. 2026 Aug 24;229(16):jeb252523. doi: 10.1242/jeb.252523 (PMC13571813; doi:10.1242/jeb.252523)
Supplement: Supplementary file 1 [file jexbio-229-252523-s1.pdf]

Table S1. ANOVA results from linear models of body mass and muscle masses

		Population	Environment	Generation	Population × Environment	Population × Generation	Environment × Generation	Population × Environment × Generation	Sex
Diaphragm	*F*	1.43	**16.8**	**8.59**	0.964	2.31	2.79	0.011	3.51
	P	0.235	**<0.001**	**0.004**	0.329	0.132	0.098	0.918	0.064
Gluteus maximus	*F*	**11.1**	**14.1**	0.618	**21.9**	**10.5**	**21.9**	**24.3**	2.96
	P	**0.001**	**<0.001**	0.435	**<0.001**	**0.002**	**<0.001**	**<0.001**	0.090
Vastus medialis	*F*	1.19	1.21	1.64	0.567	0.231	0.016	0.317	0.572
	P	0.279	0.274	0.204	0.453	0.631	0.898	0.575	0.452
Vastus lateralis	*F*	2.17	0.050	**5.18**	0.210	1.92	**4.95**	0.063	0.013
	P	0.145	0.823	**0.026**	0.648	0.170	**0.029**	0.803	0.909

F, F-value; P, P-value

Journal of Experimental Biology • Supplementary information

Table S2. Mitochondrial physiology in the different skeletal muscles in high-altitude and low-altitude populations of deer mice acclimated to warm normoxia or cold hypoxia.

	Low-altitude mice		High-altitude mice	
	**Warm normoxia**	**Cold hypoxia**	**Warm normoxia**	**Cold hypoxia**
**Diaphragm**				
Leak respiration, $L_{N,PM}$	4.63±0.25 (10)	5.22±0.28 (15)	4.87±0.32 (9)	5.30±0.21 (12)
OXPHOS respiration, P_{PM}	15.9±0.9 (10)	17.9±0.8 (15)	18.6±0.9 (9)	21.0±1.1 (12)
OXPHOS respiration, P_{PMG}	17.3±0.9 (10)	18.6±0.8 (15)	19.8±0.8 (9)	20.8±0.9 (12)
ETS respiration, E_{PMGS}	22.8±1.1 (10)	23.2±1.4 (15)	24.1±1.7 (9)	26.1±1.1 (12)
ROS emission rate, $L_{N,PM}$	2.28±0.53 (9)	2.84±0.45 (14)	2.18±0.28 (10)	1.15±0.27* (10)
ROS emission rate, P_{PM}	1.83±0.42 (9)	2.48±0.40 (14)	1.93±0.25 (10)	1.24±0.27 (10)
ROS emission rate, P_{PMG}	2.04±0.41 (9)	2.79±0.50 (14)	1.95±0.29 (10)	1.45±0.35 (10)
**Vastus medialis**				
Leak respiration, $L_{N,PM}$	3.87±0.39 (10)	4.46±0.34 (15)	4.99±0.57 (10)	4.63±0.40 (12)
OXPHOS respiration, P_{PM}	13.3±1.4 (10)	13.6±0.9 (15)	14.4±1.0 (10)	14.9±1.0 (12)
OXPHOS respiration, P_{PMG}	15.3±1.5 (10)	14.7±0.8 (15)	15.9±1.3 (10)	15.5±1.1 (12)
ETS respiration, E_{PMGS}	19.6±1.5 (10)	19.4±1.1 (15)	19.8±1.5 (10)	19.7±1.0 (12)
ROS emission rate, $L_{N,PM}$	2.54±0.83 (10)	1.68±0.29 (14)	2.76±0.60 (9)	1.11±0.18 (12)
ROS emission rate, P_{PM}	2.34±0.78 (10)	1.61±0.27 (14)	2.09±0.33 (9)	1.31±0.34 (12)
ROS emission rate, P_{PMG}	2.54±0.83 (10)	1.85±0.32 (14)	2.62±0.50 (9)	1.17±0.23 (12)
**Vastus lateralis**				
Leak respiration, $L_{N,PM}$	4.25±0.52 (10)	3.91±0.28 (14)	4.08±0.44 (10)	4.01±0.36 (11)
OXPHOS respiration, P_{PM}	13.3±1.0 (10)	12.4±0.7 (14)	13.6±1.5 (10)	14.4±0.8 (11)
OXPHOS respiration, P_{PMG}	15.3±0.9 (10)	13.6±0.8 (14)	14.8±1.6 (10)	14.4±0.9 (11)
ETS respiration, E_{PMGS}	18.7±1.0 (10)	17.7±1.0 (14)	20.7±1.9 (10)	18.6±0.9 (11)
ROS emission rate, $L_{N,PM}$	1.65±0.21 (9)	1.12±0.30 (13)	1.86±0.41 (9)	0.994±0.261 (10)
ROS emission rate, P_{PM}	1.50±0.23 (9)	1.15±0.28 (13)	1.78±0.40 (9)	1.11±0.20 (10)
ROS emission rate, P_{PMG}	1.69±0.32 (9)	1.21±0.30 (13)	2.12±0.35 (9)	1.03±0.25** (10)
**Gluteus maximus**				
Leak respiration, $L_{N,PM}$	3.83±0.56 (10)	4.03±0.30 (15)	3.95±0.46 (10)	5.09±0.66 (12)
OXPHOS respiration, P_{PM}	12.7±1.1(10)	12.8±1.0(15)	13.4±1.4 (10)	15.1±1.3 (12)
OXPHOS respiration, P_{PMG}	15.7±1.6 (10)	14.0±1.0 (15)	15.0±1.4 (10)	15.5±1.2 (12)

Journal of Experimental Biology • Supplementary information

ETS respiration, E_{PMGS}	18.9±1.7 (10)	18.8±1.7 (15)	18.2±1.6 (10)	20.5±2.2 (12)
ROS emission rate, $L_{N,PM}$	3.17±0.72 (10)	1.56±0.28** (15)	2.38±0.42 (10)	1.55±0.22 (11)
ROS emission rate, P_{PM}	2.25±0.52 (10)	1.44±0.25 (15)	2.15±0.38 (10)	1.55±0.24 (11)
ROS emission rate, P_{PMG}	3.01±0.74 (10)	1.70±0.28 (15)	2.46±0.46 (10)	1.86±0.30 (11)

Data are reported as means ± SEM for mitochondrial respiration (nmol O_2 per mg tissue per min) and ROS emission rate (pmol H_2O_2 per mg tissue per min). Measurements were made during a substrate titration protocol in non-phosphorylating (leak, *L*) conditions in the absence of adenylates (L_N), phosphorylating conditions when oxidative phosphorylation (OXPHOS) was stimulated with ADP (*P*), and the maximal capacity of the electron transport system (ETS, *E*) induced by uncoupling OXPHOS with CCCP (carbonyl cyanide m-chlorophenyl hydrazone). Substrates in each condition are shown as subscripts: P, pyruvate; M, malate; G, glutamate; S, succinate. Full ANOVA results from linear models are shown in Tables S3-S6. Holm-Sidak post-hoc tests were only performed when there were significant fixed effects in ANOVA: * significant (P<0.05) pairwise differences between highlanders and lowlanders within the same acclimation environment; ** significant (P<0.05) pairwise differences between acclimation environments within a population.

Journal of Experimental Biology • Supplementary information

Table S3. Full ANOVA results from linear models for the diaphragm

		Population	Environment	Population × Environment	Sex
Leak respiration, $L_{N,PM}$	*F*	0.215	3.64	0.076	0.076
	P	0.645	0.064	0.784	0.784
OXPHOS respiration, P_{PM}	*F*	**9.64**	**4.47**	0.0003	0.365
	P	**0.003**	**0.041**	0.986	0.549
OXPHOS respiration, P_{PMG}	*F*	**6.89**	1.62	0.035	1.19
	P	**0.012**	0.211	0.853	0.281
OXPHOS respiration, P_{PMGS}	*F*	**9.25**	2.44	0.158	0.700
	P	**0.004**	0.126	0.693	0.408
ETS respiration, E_{PMGS}	*F*	2.64	0.642	0.335	0.082
	P	0.112	0.427	0.566	0.776
Complex IV respiration, P_{Tm}	*F*	**6.59**	**9.01**	0.079	0.017
	P	**0.014**	**0.004**	0.779	0.897
P_{PM}/P_{PMGS}	*F*	0.361	0.755	0.272	0.019
	P	0.551	0.390	0.604	0.892
P_{PMG}/P_{PMGS}	*F*	0.283	0.413	0.429	0.340
	P	0.597	0.524	0.516	0.563
E_{PMGS}/ P_{PMGS}	*F*	1.00	0.575	2.79	0.131
	P	0.323	0.453	0.102	0.720
Volume density of mitochondria per fibre	*F*	0.764	**7.70**	0.061	0.809
	P	0.393	**0.012**	0.808	0.379
Volume density of subsarcolemmal mitochondria per fibre	*F*	0.708	**15.9**	0.258	2.30
	P	0.410	**<0.001**	0.618	0.145
Volume density of intermyofibrillar mitochondria per fibre	*F*	0.224	0.272	0.080	0.269
	P	0.641	0.608	0.780	0.610
Proportion of subsarcolemmal mitochondria	*F*	0.605	**13.9**	0.774	2.67
	P	0.446	**0.001**	0.390	0.119
Capillary density	*F*	0.977	**7.15**	0.056	0.863
	P	0.335	**0.015**	0.815	0.364
Capillary to fibre ratio	*F*	0.290	**24.6**	1.29	0.020
	P	0.596	**<0.001**	0.270	0.888
Numerical density of oxidative fibres	*F*	1.14	0.717	1.15	0.781
	P	0.298	0.408	0.297	0.388
Numerical density of glycolytic fibres	*F*	0.527	1.03	1.46	0.979
	P	0.477	0.324	0.241	0.334
ROS emission rate, $L_{N,PM}$	*F*	**5.46**	0.223	3.99	1.59
	P	**0.025**	0.640	0.053	0.215
ROS emission rate, P_{PM}	*F*	3.18	0.001	3.56	1.02
	P	0.082	0.973	0.067	0.319
ROS emission rate, P_{PMG}	*F*	3.59	0.142	2.47	2.62
	P	0.065	0.708	0.124	0.114
ROS emission rate, P_{PMGS}	*F*	1.62	1.08	**5.88**	0.805
	P	0.211	0.304	**0.020**	0.375
MitoP/MitoB	*F*	<0.001	0.013	0.789	0.176
	P	0.990	0.905	0.382	0.678

F, F-value; P, P-value. See Table S2 for other abbreviations.

Table S4. Full ANOVA results from linear models for vastus medialis

		Population	Environment	Population × Environment	Sex
Leak respiration, $L_{N,PM}$	*F*	1.81	0.102	1.22	0.293
	P	0.186	0.752	0.276	0.591
OXPHOS respiration, P_{PM}	*F*	1.13	0.130	0.005	0.877
	P	0.293	0.720	0.944	0.354
OXPHOS respiration, P_{PMG}	*F*	0.427	0.217	0.004	1.18
	P	0.517	0.643	0.950	0.283
OXPHOS respiration, P_{PMGS}	*F*	1.21	0.070	0.154	0.116
	P	0.277	0.792	0.696	0.735
ETS respiration, E_{PMGS}	*F*	0.178	0.014	0.032	0.083
	P	0.676	0.908	0.857	0.774
Complex IV respiration, P_{Tm}	*F*	**4.32**	**6.21**	0.379	0.125
	P	**0.044**	**0.017**	0.541	0.726
P_{PM}/P_{PMGS}	*F*	0.108	0.978	1.03	0.759
	P	0.744	0.328	0.316	0.389
P_{PMG}/P_{PMGS}	*F*	1.33	0.520	2.26	3.92
	P	0.256	0.475	0.140	0.054
E_{PMGS}/ P_{PMGS}	*F*	2.23	0.510	1.30	2.83
	P	0.143	0.479	0.260	0.100
Volume density of mitochondria per fibre	*F*	0.004	**7.62**	0.153	0.361
	P	0.951	**0.012**	0.700	0.555
Volume density of subsarcolemmal mitochondria per fibre	*F*	1.08	2.57	0.042	0.168
	P	0.312	0.125	0.840	0.686
Volume density of intermyofibrillar mitochondria per fibre	*F*	1.53	**8.23**	0.769	2.11
	P	0.231	**0.010**	0.391	0.162
Proportion of subsarcolemmal mitochondria	*F*	3.74	0.113	0.807	0.974
	P	0.068	0.741	0.380	0.336
Capillary density	*F*	0.705	**6.25**	0.459	**4.72**
	P	0.411	**0.022**	0.506	**0.043**
Capillary to fibre ratio	*F*	0.141	**6.12**	0.291	0.157
	P	0.711	**0.023**	0.698	0.698
Numerical density of oxidative fibres	*F*	0.390	3.37	1.26	0.091
	P	0.540	0.082	0.276	0.766
Numerical density of glycolytic fibres	*F*	0.390	3.37	1.26	0.091
	P	0.540	0.082	0.276	0.766
ROS emission rate, $L_{N,PM}$	*F*	0.205	**6.08**	0.622	0.543
	P	0.653	**0.018**	0.435	0.465
ROS emission rate, P_{PM}	*F*	0.363	2.76	0.002	0.037
	P	0.550	0.104	0.962	0.848
ROS emission rate, P_{PMG}	*F*	0.495	**4.53**	0.566	0.416
	P	0.486	**0.039**	0.456	0.522
ROS emission rate, P_{PMGS}	*F*	0.125	*3.87*	1.50	0.297
	P	0.726	*0.056*	0.227	0.588
MitoP/MitoB	*F*	**4.76**	**4.78**	0.523	0.494
	P	**0.038**	**0.038**	0.476	0.488

F, F-value; P, P-value. See Table S2 for other abbreviations.

Journal of Experimental Biology • Supplementary information

Table S5. Full ANOVA results from linear models for vastus lateralis

		**Population**	**Environment**	**Population × Environment**	**Sex**
Leak respiration, $L_{N,PM}$	*F*	0.0007	0.272	0.115	0.020
	P	0.980	0.605	0.736	0.889
OXPHOS respiration, P_{PM}	*F*	1.619	0.017	0.778	0.244
	P	0.211	0.896	0.383	0.624
OXPHOS respiration, P_{PMG}	*F*	0.084	0.882	0.336	0.014
	P	0.774	0.353	0.565	0.907
OXPHOS respiration, P_{PMGS}	*F*	1.16	1.53	<0.001	0.021
	P	0.288	0.223	0.996	0.884
ETS respiration, E_{PMGS}	*F*	1.41	1.43	0.174	0.164
	P	0.243	0.239	0.679	0.687
Complex IV respiration, P_{Tm}	*F*	**4.70**	0.492	1.54	0.704
	P	**0.036**	0.487	0.221	0.406
P_{PM}/P_{PMGS}	*F*	0.137	2.23	2.21	0.494
	P	0.713	0.135	0.145	0.486
P_{PMG}/P_{PMGS}	*F*	2.89	0.011	2.22	0.231
	P	0.097	0.915	0.144	0.633
E_{PMGS}/ P_{PMGS}	*F*	0.020	0.035	0.548	0.516
	P	0.887	0.852	0.464	0.477
ROS emission rate, $L_{N,PM}$	*F*	0.043	**4.84**	0.289	0.198
	P	0.837	**0.034**	0.594	0.659
ROS emission rate, P_{PM}	*F*	0.202	2.87	0.293	0.133
	P	0.655	0.099	0.591	0.717
ROS emission rate, P_{PMG}	*F*	0.187	**6.16**	0.927	1.12
	P	0.668	**0.018**	0.342	0.296
ROS emission rate, P_{PMGS}	*F*	0.291	1.79	3.35	0.095
	P	0.593	0.189	0.075	0.759
MitoP/MitoP	*F*	0.270	**4.22**	0.014	1.36
	P	0.607	**0.049**	0.907	0.254

F, F-value; P, P-value. See Table S2 for other abbreviations.

Journal of Experimental Biology • Supplementary information

Table S6. Full ANOVA results from linear models for gluteus maximus

		Population	Environment	Population × Environment	Sex
Leak respiration, $L_{N,PM}$	*F*	1.56	1.66	0.847	0.623
	P	0.219	0.205	0.363	0.434
OXPHOS respiration, P_{PM}	*F*	1.66	0.547	0.429	0.619
	P	0.204	0.463	0.516	0.436
OXPHOS respiration, P_{PMG}	*F*	0.262	0.171	0.541	0.156
	P	0.611	0.681	0.466	0.694
OXPHOS respiration, P_{PMGS}	*F*	0.071	0.423	0.376	0.0004
	P	0.791	0.519	0.543	0.985
ETS respiration, E_{PMGS}	*F*	0.083	0.315	0.357	0.136
	P	0.775	0.577	0.553	0.714
Complex IV respiration, P_{Tm}	*F*	0.014	1.01	0.728	0.242
	P	0.905	0.320	0.398	0.625
P_{PM}/P_{PMGS}	*F*	**4.31**	**8.72**	0.024	2.29
	P	**0.044**	**0.005**	0.878	0.138
P_{PMG}/P_{PMGS}	*F*	1.73	1.02	0.097	2.20
	P	0.195	0.319	0.757	0.146
E_{PMGS}/ P_{PMGS}	*F*	0.009	**5.06**	0.003	0.571
	P	0.925	**0.030**	0.954	0.454
ROS emission rate, $L_{N,PM}$	*F*	**5.46**	0.223	3.99	1.59
	P	**0.025**	0.640	0.053	0.215
ROS emission rate, P_{PM}	*F*	3.18	0.001	3.56	1.02
	P	0.082	0.973	0.067	0.319
ROS emission rate, P_{PMG}	*F*	3.59	0.142	2.47	2.62
	P	0.065	0.708	0.124	0.114
ROS emission rate, P_{PMGS}	*F*	0.539	**5.99**	0.516	0.081
	P	0.467	**0.019**	0.477	0.777
MitoP/MitoP	*F*	0.046	**4.42**	0.136	0.325
	P	0.832	**0.045**	0.715	0.574

F, F-value; P, P-value. See Table S2 for other abbreviations.

Journal of Experimental Biology • Supplementary information
